# Assessment of General Population Understanding and Awareness of Drug Addiction before and after a Health Educational Campaign in Taif City, Saudi Arabia

**DOI:** 10.3390/healthcare12181877

**Published:** 2024-09-19

**Authors:** Mohammad S. Alzahrani, Huriyyah A. Alotaibi, Shahad M. Alhumayani, Hadeel F. Aljuaid, Refah S. Alghamdi, Yusuf S. Althobaiti

**Affiliations:** 1 Department of Clinical Pharmacy, College of Pharmacy, Taif University, P.O. Box 11099, Taif 21944, Saudi Arabia; m.s.alzahrani@tu.edu.sa; 2 Addiction and Neuroscience Research Unit, Taif University, P.O. Box 11099, Taif 21944, Saudi Arabia; 3 Department of Pharmacology and Toxicology, College of Pharmacy, Taif University, P.O. Box 11099, Taif 21944, Saudi Arabia

**Keywords:** substance use disorders, knowledge, attitude, awareness, Saudi Arabia

## Abstract

Objectives: An awareness campaign was carried out in Taif City to increase awareness about substance abuse and its dangers. This study sought to evaluate the effectiveness of the campaign in enhancing the study participants’ awareness and knowledge about drug addiction. Methods: Using pre- and post-campaign surveys, we assessed the campaign’s impact on participants’ understanding of drug addiction. The survey included demographic items, followed by 12 items for awareness, rated on a 5-point Likert scale, with the total scores ranging up to 60. Results: A total of 146 visitors, with a mean age of 33.4 years (SD = 9.2), completed both the pre- and post-questionnaires. Notably, in the post-campaign, 47.3% of the participants acknowledged the risk of IV drug-related infections, a substantial increase from 28.1% from the pre-campaign. Overall, the mean total awareness score increased significantly, from 47.4 (SD = 6.6) pre-campaign to 50.4 (SD = 6.4) post-campaign (paired t = −4.052, *p* < 0.001). Conclusion: These findings highlight the potential of such campaigns to significantly improve understanding and awareness of drug addiction.

## 1. Introduction

Substance abuse is a complex cognitive, behavioral, and psychological disorder. This disorder is a compulsive and chronic relapsing mental disorder that is characterized by an uncontrollable desire to seek drugs, a lack of capacity to limit consumption, and the emergence of a withdrawal syndrome during the cessation of drug use despite an awareness of the harmful consequences [1]. As one of the most antisocial behaviors, the reasons for the abuse of drugs are complex and vary by geographic region and demographic characteristics. Of note, the underlying causes of drug abuse are diverse, such as peer pressure, depression, curiosity, attempts to increase or improve performance, hedonism, alienation, rebellion, and a wide range of other causes, with substance abuse ranging from the use of solvents to stimulants and opiates [2]. Interestingly, drug abuse-related deaths have significantly increased over the past decade. These increases have represented a growing public health concern. Naturally, this has given rise to intense debate regarding the merits of intervention policies designed to limit drug abuse and its consequences [3]. 

According to a clinical record review in Saudi Arabia that was published in 2019, there was a positive increase in admissions cases in Al Amal Hospital of Dammam between 1993 and 2013 associated with substance abuse [4]. The main factors that changed in 2013 for first-time admission included having a criminal record, being single, being unemployed, cannabis and alcohol abuse, being a youth, and being middle-aged [4]. 

Preventive measures, such as laws, public awareness campaigns, and university-based education initiatives, have been suggested as potentially valuable techniques to assist in decreasing substance abuse progression. Utilizing past knowledge of drug abuse prevention initiatives that were successful, it is possible that a lot of people could benefit from public education campaigns to aid in changing the perceptions of substance usage and dependence. In order to help reduce drug consumption, a comprehensive prevention effort must be launched, given the catastrophic repercussions of the substance abuse epidemic [5]. Accordingly, we aimed to conduct an awareness campaign in Taif City to increase awareness about substance abuse and its dangers. To achieve maximum community awareness, the “Together to Protect Them” campaign was an on-site activity led by the Deanship of Community Service and Sustainable Development at Taif University in cooperation with the General Directorate of Narcotics Control and KAFA Charitable Society. This program addressed the need to educate the Taif community on the concerns of substance abuse and addiction.

The “Together to Protect Them” campaign is the subject of this research project. We sought to evaluate the effectiveness of the campaign regarding participants’ understanding and awareness of drug addiction. The necessity of this study stems from the growing public health concern surrounding drug addiction and the urgent need for effective awareness campaigns. Despite numerous interventions, gaps remain in public understanding and prevention strategies. Our study aims to address these gaps by evaluating the impact of a targeted awareness campaign on participants’ knowledge and awareness of drug addiction. By focusing on this specific intervention, we seek to provide evidence of its effectiveness and offer insights into how similar campaigns can be optimized to better serve the community.

## 2. Materials and Methods

### 2.1. Study Design and Setting

A pre- and post-study was conducted to evaluate the effectiveness of the “Together to Protect Them” campaign. The campaign was held on-site in November 2022 at Jouri Mall, one of the largest malls in Taif City, Saudi Arabia. Around 300 people attended the event. Study participants were recruited through a non-probability sampling technique, where visitors who consented to participate in the study were interviewed and asked to fill out both the pre- and post-questionnaires to measure the extent of their awareness and the effectiveness of the campaign in achieving its goals. All participants were informed about the study’s purpose and assured of the anonymity of their responses. Identifiers were not collected on the survey, and informed consent was collected before participation. The Scientific Research Ethics Committee at Taif University reviewed and approved this study.

### 2.2. Survey Tool

The survey included demographics, such as age, gender, and the highest education level attained. The survey also included a question about whether the participants had ever dealt with people with drug addiction problems, followed by 12 items for awareness of drug addiction. These items were intended to assess the participants’ knowledge about drug addiction, recovery ways, prophylaxis of substance abuse, and the role of the family. The 12 items were rated on a 5-point Likert scale, ranging from “(1) strongly disagree” to “(5) strongly agree”. The points were added together to calculate the awareness score for each respondent, with a maximum score of 60. The post-survey contained an additional item about the effectiveness of the campaign. The survey was evaluated for face validity by two faculty members who had knowledge of the subject. The survey was then pilot-tested on a small number of subjects to clarify the items. 

### 2.3. Statistical Analysis

In this study, univariable and bivariable analyses were utilized. The mean and median were calculated for the numerical variables. The frequency and percentages were calculated for the categorical variables. Pre- and post-awareness scores were compared using a paired *t*-test to assess the extent of improvements in awareness. The differences in awareness scores by characteristics were assessed using a Student’s t-test or one-way ANOVA. Statistical significance was set a priori at *p* < 0.05. All statistical analyses were performed using SPSS software ver. 22.0 (IBM, Armonk, NY, USA). 

## 3. Results

A total of 146 visitors completed both the pre- and post-campaign questionnaires. Based on the demographic characteristics, the gender distribution among the respondents was 54 (37%) females and 90 (61.6%) males. The mean age was 33.4 years old (SD = 9.2 years). As for the educational level, 69 (47.3%) participants achieved a bachelor’s degree, 49 (33.6%) were high school graduates, 21 (14.4%) were master’s holders, and 5 (3.4%) were Ph.D. holders. Regarding the frequency of dealing with people with an addiction, 91 (62.3%) participants had not ever dealt with them, whereas 53 (36.3%) reported dealing with them. 

More than three-quarters of the participants (77.4%) strongly agreed that the campaign was beneficial to them, and a further 17.1% also agreed, so there were only four participants (2.7%) who were neutral to the benefit of the campaign and one (0.1%) respondent indicated their strong disagreement with the campaign.

The changes in understanding toward drug addiction before and after attending the campaign are shown in Table 1. There were 54 (37%) participants who strongly agreed prior to the campaign that ‘addiction is a degenerative disease that affects the mind due to the continuous use of narcotic substances.’ However, this number increased to 71 (48.6%) after the campaign. Knowledge about ‘withdrawal symptoms’ improved from 42 (28.8%) before the campaign to 61 (41.8%) after the campaign, and the number of participants who were ‘neutral’ to the definition of withdrawals dropped because of attendance at the campaign from 27 (18.5%) to 21 (14.4%).

Twenty-one participants (14.4%) strongly agreed before the campaign that opioids affect the gastrointestinal system by causing constipation and types of cancer, including cancers of the stomach, colon, rectum, and esophagus. Their number increased to 52 (35.6%) after the educational campaign. The risk of infections related to IV drug use was acknowledged by 69 (47.3%) of the participants, compared to 41 (28.1%) before the campaign.

Only 17 (11.6%) participants strongly noted that cocaine and amphetamines were not narcotics prior to the campaign, with the number rising to 24 (16.4%) afterward. Similarly, the number of those strongly noting that morphine and heroin are not stimulant drugs rose from 5 (3.4%) to 15 (10.3%). The link between dopamine and addiction was spotted by 63 (43.2%) participants after the campaign, compared to only 35 (24%) before it. There were 60 (41.1%) participants prior to the campaign who believed that relapse prevention can be achieved by seeing a psychiatrist, going to support meetings, and taking prescribed medications. After the campaign, 75 (51.4%) participants appreciated the effectiveness of these ways. 

The family role was emphasized by 89 (61%) participants prior to campaigning and by 110 (75.3%) following it. With the campaign, 108 (74%) participants saw the effectiveness of role modeling and enhanced communication in universal prevention of substance use (compared to 90 (61.6%) participants prior to the campaign). Furthermore, nearly half of the participants, 81 (55.5%), agreed that addiction risk could be prevented by not taking the substances at all; their number increased to 114 (78.1%) after the campaign. Post-treatment support was strongly seen as a requirement among 91 (62.3%) participants pre-campaign and rose to 105 (71.9%) post-campaign.

**Figure 1:**
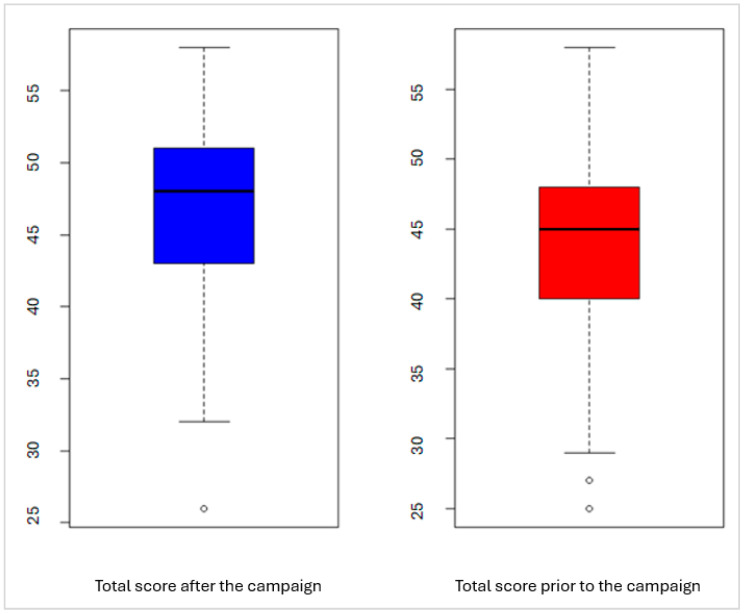
Change in participants’ total awareness scores towards substance use before and after the campaign.

Prior to the campaign, the mean total score was 47.4 points (SD = 6.6 points). In contrast, the post-campaign total score mean was higher at 50.4 points (SD = 6.4 points). This difference was statistically significant (paired t = −4.052, *p* < 0.001) (Figure 1).

The mean awareness score among the male participants was slightly higher than that of the female participants, both before and after the campaign. However, this difference was not statistically significant (Table 2). Similarly, there was no statistically significant difference in the mean awareness scores in terms of age, gender, and education levels (*p*-value > 0.05). The participants who reported they had ever dealt with people with drug addictions had a slightly higher average awareness score than those who had never dealt with people with drug addictions, both pre- and post-campaign. 

## 4. Discussion

The primary purpose of this study was to evaluate the impact of an awareness campaign on the knowledge and attitudes of participants toward drug addiction. According to the results of our survey, the visitors were generally aware of the harmful effects of addiction and the nature of substance use and had a relatively good knowledge level. In our Islamic country, alcohol and substance use is forbidden. Additionally, the substantial social stigma associated with substance use is a significant cause that prevents people with an addiction from seeking help. To break that social stigma, we need to bring this dialog into the public domain as the first step and form a clear and directed health message [1]. However, 7–8% of Saudis reported using drugs, and 70% of all drug users were between the ages of 12 and 22 [6]. This age group falls within the adolescence period, which is considered a crucial time in a person’s life and is often regarded as the most transformational stage [7]. 

Substance abuse is more prevalent among adolescents. Adolescence is a time of change on all levels—physical, emotional, social, and psychological—which makes them more at risk of drug addiction as a result of these changes. Adolescents are more likely than those of other ages to use psychoactive substances or illicit drugs because they are more likely to separate from their parents, make independent decisions, act like adults, and confirm their identity [8]. A previous study showed that the age range between 18 and 24 is the one during which most addiction to narcotics is prevalent [9].

Many studies have discussed the factors that prompt young people to use illegal drugs. According to a Nigerian study, peer pressure, emotional issues, relationship issues, media, youthful exuberance, the desire to feel “high”, and other factors are all thought to have an impact on teenagers using illegal drugs [10]. Similarly, a study conducted in Bangladesh found that the prevalence of substance use may increase due to several factors, such as higher availability of the substances, peer influence, more pocket money, being influenced by seeing others’ engagements for fun or partying, and family negligence [11]. A study from Al Qassim looked at the impact of other factors on addictive behavior, including unemployment and single status. Being unemployed and unmarried leads to extreme stress, rejection, fear, and depressive episodes, which are major risk factors for developing a drug use issue. Unemployed and unmarried people frequently use alcohol and drugs to alleviate their discomfort and sense of helplessness [12]. Regarding educational levels, a low educational level has a negative impact on health and promotes drug usage [13,14]. Education has a role in helping people acquire knowledge and form risk perceptions. According to a study conducted in Woodlawn, the least educated individuals are more likely to have drug use disorders [15].

A substantial improvement was noted before and after the campaign with regard to participants’ knowledge about drug addiction. Prior to the campaign, 21 (14.4%) participants firmly believed that opioids had an adverse effect on the gastrointestinal system by inducing constipation and various malignancies, such as cancers of the stomach, colon, rectum, and esophagus. After the instructional effort, their number increased to 52 (35.6%). Compared to 41 (28.1%) participants before the campaign, 69 (47.3%) individuals recognized the risk of infections associated with IV drug use. Prior to the campaign, only 17 (11.6%) people strongly agreed that cocaine and amphetamines are not narcotics; after the campaign, that number increased to 24 (16.4%). Similarly, from 5 (3.4%) people to 15 (10.3%) people strongly agreed that morphine and heroin are not stimulant substances. Sixty-three people (43.2%) recognized the connection between dopamine and addiction after the campaign, as opposed to 35 (24%) before the campaign. Prior to the campaign, 60 (41.1%) people thought that seeing a psychiatrist, attending support groups, and taking prescription drugs may help avoid relapses. After the campaign, 75 (51.4%) people agreed that these methods were successful. 

Our study revealed a significant rise in the number of participants who underscored the crucial role of family support in combating drug addiction. Following the campaign, the proportion of such participants surged from 61% (89 individuals) to a noteworthy 75.3% (110 individuals). This indicated an enhanced awareness of the family’s pivotal role in overcoming drug addiction. Families in today’s society encounter a variety of difficulties, including marital issues, poverty, the absence of one parent, and poor parent–child communication, all of which can have detrimental impacts on children. As a result, these difficulties may disrupt and weaken family support, which increases the likelihood of using drugs [16]. The family’s support and strong emotional connections may protect them from developing high-risk behaviors, norm-breaking tendencies, and, ultimately, substance abuse.

A previous study suggested a link between parental status and higher levels of addiction [17]. Participants with a parent who has passed away or who have parents who are divorced seem to have a higher addiction profile than participants whose parents are still together. This can be explained by the higher levels of stress and adversity that are present in broken homes as opposed to intact ones, which are more likely to push young adolescents to engage in risky behaviors like smoking and binge drinking as potential coping mechanisms. Young people may receive less support from their broken or nonexistent homes and turn to these habits as their only means of coping with their issues. Another study found that families that have an adolescent who is addicted to drugs or alcohol have higher couple scores on depression, anxiety, and stress, as well as lower marital satisfaction and family quality of life [18]. According to the study, there is a bidirectional relationship between family dynamics and adolescent substance addiction in Arab families. Family issues may result in adolescent substance abuse, and adolescent substance addiction may also have an impact on parents’ mental health and the quality of their marriage. 

Following the campaign, a substantial majority of the participants, approximately 74%, came to appreciate the significance of role models in enhancing communication strategies for universal drug use prevention. This marked a notable increase from the pre-campaign figure of 61.6%. Furthermore, the campaign led to a shift in participants’ attitudes towards drug use. More than half of the participants concurred that abstaining from drug use could potentially mitigate the risk of addiction. Post-campaign, this sentiment was echoed by an even larger group, with 114 participants, accounting for 78.1% of the participants, endorsing this view. Furthermore, prior to the campaign, 91 participants (62.3%) were in strong agreement about the necessity of post-treatment support. This underscores the campaign’s effectiveness in shifting perceptions and attitudes toward drug use and recovery.

As mentioned above, due to prevalent Islamic cultural and societal limitations, drug users may find it difficult to admit to abusing drugs, which makes it more difficult to conduct studies that could reveal the prevalence of drug use in the general community. Therefore, there can potentially be a sizable quantity of abuse that is concealed in Saudi Arabia [19]. Another study stated and agreed on the importance of these substance use health education campaigns in raising public awareness and demonstrating how much the community needs them [20], besides the need for future studies that will improve the strategies of drug addiction [21]. Several studies have evaluated similar awareness campaigns in different communities. A previous study conducted in the United States assessed the impact of a drug awareness campaign on high school students and found a significant reduction in youth substance use [22]. Similarly, a campaign in Australia targeting young adults demonstrated that tailored messages significantly improved awareness and understanding of the risks associated with drug addiction [23]. These examples highlight the diverse approaches and positive outcomes of drug awareness campaigns across various demographics and regions, underscoring the potential impact of our campaign.

To translate our findings into real-world clinical practice, we recommend integrating educational campaigns similar to the one conducted in this study into routine clinical practice. These campaigns can significantly enhance patients’ and their families’ awareness and understanding of drug addiction and its prevention. Additionally, developing and implementing family-centered care models that actively involve family members in the treatment and recovery process can be beneficial. Regularly assessing the effectiveness of these educational interventions and adapting them based on feedback and changing community needs will ensure their continued relevance and impact. By adopting these strategies, healthcare providers can bridge the gap between research and clinical practice, ultimately improving patient outcomes. 

This study, while offering valuable insights, does come with a few notable limitations that warrant careful consideration. Firstly, the absence of a control or comparison group can pose a threat to internal validity and limit our ability to account for potential confounding factors and historical events. Secondly, the use of self-reported questionnaires as a primary data collection method introduces the potential for recollection bias, as participants’ memories of events may not always be accurate or complete. Thirdly, the generalizability of our findings is somewhat limited due to the use of convenience sampling. In addition, the number of female participants was lower than that of male participants. This gender imbalance may also affect the generalizability of our findings. These factors should be kept in mind when interpreting the results of this study.

## 5. Conclusions

Our finding suggests that the awareness campaign “Together to Protect Them” has fulfilled its goal of enhancing understanding and awareness toward drug addiction. Future health education campaigns may consider employing a multi-model approach with a larger focus on supported drug addiction treatment programs and a dedication to reaching out to a wider range of the community. Future research should also assess the abilities and dispositions of medical personnel toward patients who abuse drugs, as well as the protocols that should be followed. They must coordinate efforts and provide assistance to Taif residents and other cities who are dealing with behavioral problems.

## Figures and Tables

**Table 1:** Changes in participants’ awareness toward substance use before and after the campaign.

Item	Time	Strongly Agree	Agree	Neutral	Disagree	Strongly Disagree
1/Addiction is a degenerative disease that affects the mind due to the continuous use of narcotic substances. A person becomes dependent on them and needs to increase the dose from time to time to get the same effect.	Pre	54 (37%)	55 (37.7%)	23 (15.8%)	9 (6.2%)	5 (3.4%)
	Post	71 (48.6%)	46 (31.5%)	21 (14.4%)	1 (0.7%)	7 (4.8%)
2/In the case of discontinuing drug use, the addict will develop serious psychological and physical symptoms called withdrawal symptoms that may lead to death.	Pre	42 (28.8%)	60 (41.1%)	27 (18.5%)	14 (9.6%)	3 (2.1%)
	Post	61 (41.8%)	50 (34.2%)	21 (14.4%)	8 (5.5%)	6 (4.1%)
3/Opioids reduce bowel movements, causing constipation. Long-term effects may be associated with the occurrence of various cancers, including cancers of the stomach, colon, rectum, and esophagus.	Pre	21 (14.4%)	51 (34.9%)	68 (46.6%)	4 (2.7%)	2 (1.4%)
	Post	52 (35.6%)	46 (31.5%)	41 (28.1%)	4 (2.7%)	3 (2.1%)
4/The risks of using intravenous (injecting) drugs are bacterial infection in the blood vessels, infections of the valves and the lining of the heart, and clots.	Pre	41 (28.1%)	59 (40.4%)	43 (29.5%)	2 (1.4%)	1 (0.7%)
	Post	69 (47.3%)	53 (36.3%)	19 (13%)	2 (1.4%)	3 (2.1%)
5/Cocaine and amphetamines are classified as narcotic analgesics.	Pre	15 (10.3%)	29 (19.9%)	65 (44.5%)	20 (13.7%)	17 (11.6%)
	Post	24 (16.4%)	34 (23.3%)	39 (26.7%)	25 (17.1%)	24 (16.4%)
6/Morphine and heroin are classified as stimulant drugs.	Pre	27 (18.5%)	42 (28.8%)	61 (41.8%)	11 (7.5%)	5 (3.4%)
	Post	42 (28.8%)	47 (32.2%)	32 (21.9%)	10 (6.8%)	15 (10.3%)
7/Drug addiction occurs due to an increase in the level of dopamine in the body and creates a state of euphoria, happiness, and temporary pleasure.	Pre	35 (24%)	48 (32.9%)	55 (37.7%)	6 (4.1%)	2 (1.4%)
	Post	63 (43.2%)	46 (31.5%)	31 (21.2%)	6 (4.1%)	0 (0%)
8/The ways to prevent relapse are to see a psychiatrist, go to support group meetings, or take prescribed medication.	Pre	60 (41.1%)	51 (34.9%)	30 (20.5%)	2 (1.4%)	3 (2.1%)
	Post	75 (51.4%)	57 (39%)	10 (6.8%)	2 (1.4%)	2 (1.4%)
9/The family has a role in substance abuse prevention.	Pre	89 (61%)	45 (30.8%)	6 (4.1%)	3 (2.1%)	3 (2.1%)
	Post	110 (75.3%)	31 (21.2%)	3 (2.1%)	2 (1.4%)	0 (0%)
10/One of the ways to prevent the risk of addiction is not to take drugs at all, even in small doses or just for the experience.	Pre	81 (55.5%)	33 (22.6%)	22 (15.1%)	6 (4.1%)	4 (2.7%)
	Post	114 (78.1%)	21 (14.4%)	7 (4.8%)	2 (1.4%)	2 (1.4%)
11/Good role models, communication, and listening are ways to prevent children from abusing substances.	Pre	90 (61.6%)	38 (26%)	12 (8.2%)	4 (2.7%)	2 (1.4%)
	Post	108 (74%)	34 (23.3%)	4 (2.7%)	0 (0%)	0 (0%)
12/There is a need to provide a supportive environment for the addict after completing the treatment stages.	Pre	91 (62.3%)	38 (26%)	11 (7.5%)	4 (2.7%)	2 (1.4%)
	Post	105 (71.9%)	39 (26.7%)	1 (0.7%)	1 (0.7%)	0 (0%)

**Table 2:** Differences in the mean awareness score by characteristics, pre- and post-campaign.

Characteristic	Mean Awareness Score ± SD	
	Pre-Campaign	Post-Campaign
***Gender***		
Female	46.9 ± 5.2	49.5 ± 5.2
Male	47.4 ± 7.3	50.9 ± 6.9
*p-value*	0.662	0.222
***Age***		
34 years or younger	46.6 ± 6.8	50.1 ± 6.6
35 years or older	47.9 ± 6.3	50.8 ± 6.2
*p-value*	0.215	0.222
***Highest Education***		
High school	46.9 ± 6.4	49.7 ± 7.6
Bachelor	47.2 ± 7.1	50.7 ± 5.9
Master	47.7 ± 6.5	50.6 ± 5.4
PhD	49.6 ± 1.7	50.8 ± 5.4
*p-value*	0.842	0.813
***Ever dealt with drug addicts***		
No	46.8 ± 6.7	49.7 ± 6.2
Yes	47.9 ± 6.4	51.5 ± 6.7
*p-value*	0.346	0.129

## Data Availability

All data generated or analyzed during this study are included in this published article.
